# Intermittent Preventive Treatment to Reduce the Burden of Malaria in Children: New Evidence on Integration and Delivery

**DOI:** 10.1371/journal.pmed.1000410

**Published:** 2011-02-01

**Authors:** James G. Beeson, Stephen J. Rogerson, Ivo Mueller, Jack S. Richards, Freya J. I. Fowkes

**Affiliations:** 1 The Walter and Eliza Hall Institute of Medical Research, Parkville, Victoria, Australia; 2 Department of Medicine, University of Melbourne, Parkville, Victoria, Australia; 3 Barcelona Centre for International Health Research (CRESIB), Barcelona, Spain; 4 Papua New Guinea Institute of Medical Research, Goroka, Papua New Guinea

## Abstract

James Beeson and colleagues discuss three new studies in *PLoS Medicine* that provide valuable evidence on how to delivery and integrate intermittent preventive reatment for malaria in children (IPTc).

**Linked Research Articles**

This Perspective discusses the following new studies published in *PLoS Medicine*:

1. Dicko A, Diallo AI, Tembine I, Dicko Y, Dara N, et al. (2011) Intermittent Preventive Treatment of Malaria Provides Substantial Protection against Malaria in Children Already Protected by an Insecticide-Treated Bednet in Mali: A Randomised, Double-Blind, Placebo-Controlled Trial. PLoS Med 8(2): e1000407. doi:10.1371/journal.pmed.1000407.

A randomized trial reported by Alassane Dicko and colleagues shows that intermittent preventive treatment for malaria in children who are protected from mosquitoes using insecticide-treated bednets provides substantial protection from malaria.

2. Konaté AT, Yaro JB, Ouédraogo AZ, Diarra A, Gansané A, et al. (2011) Intermittent Preventive Treatment of Malaria Provides Substantial Protection against Malaria in Children Already Protected by an Insecticide-Treated Bednet in Burkina Faso: A Randomised, Double-Blind, Placebo-Controlled Trial. PLoS Med 8(2): e1000408. doi:10.1371/journal.pmed.1000408.

A randomized trial reported by Diadier Diallo and colleagues shows that intermittent preventive treatment for malaria in children who are protected from mosquitoes using insecticide-treated bednets provides substantial protection from malaria.

3. Bojang KA, Akor F, Conteh L, Webb E, Bittaye O, et al. (2011) Two Strategies for the Delivery of IPTc in an Area of Seasonal Malaria Transmission in The Gambia: A Randomised Controlled Trial. PLoS Med 8(2): e1000409. doi:10.1371/journal.pmed.1000409.

Kalifa Bojang and colleagues report a randomized trial showing that delivery of intermittent preventive treatment for malaria in children by village health workers is more effective than delivery by reproductive and child health trekking clinics.

Over the past decade, there has been a substantial increase globally in the coverage of effective interventions to reduce morbidity and mortality from malaria. Insecticide-treated bed nets (ITNs), indoor residual spraying with insecticides, and the use of more effective antimalarials have been the cornerstones of such measures, and there are now clear signs of a sustained reduction in the burden of malaria in many regions. However, further interventions are urgently needed to capitalise on recent progress and sustain the malaria control and elimination effort.

## Intermittent Preventive Treatment of Malaria

One approach to help reduce the burden of malaria caused by *Plasmodium falciparum* is intermittent preventive treatment (IPT), which involves periodic therapeutic doses of antimalarials to reduce the incidence of malaria and prevalence of anemia [1],[2]. Previous efforts to use sustained chemoprophylaxis to prevent malaria were complicated by drug toxicity and safety concerns, promoting drug resistance, rebound increases in malaria once chemoprophylaxis ended, and logistical challenges in implementing such an intervention at the population level [3]. Instead, IPT seeks to balance these factors by providing treatments that are frequent enough to have significant benefit, without the downsides of chemoprophylaxis. It appears to act in part by post-treatment prophylactic effects [4], while drug levels remain above inhibitory concentrations; long-acting drugs such as sulfadoxine-pyrimethamine (SP) and amodiaquine (AQ) have been widely used. IPT in pregnancy (IPTp) reduces anemia and low birth weight [5], and is now recommended by the World Health Organization (WHO) for all pregnant women at risk in sub-Saharan Africa. IPT in infants (IPTi) decreases malaria episodes by 22%–59% [6]. Since malaria is a major cause of illness and death of children under 5 years [7], recent studies have evaluated the potential for using IPT during childhood (known as IPTc) [8]–[10], including three new papers in *PLoS Medicine* that add to the growing evidence base [11]–[13].

## New Evidence for IPTc in the Context of Established Interventions

IPTc appears to be highly effective at reducing clinical malaria episodes in children under 5 with protective efficacy against symptomatic malaria of 69% or higher [8]–[10]. Two new trials add to our understanding by studying the efficacy of IPTc in the context of established interventions. These trials in Burkina Faso and Mali, published in *PLoS Medicine*, now show that the protective efficacy of IPTc extends to children sleeping under ITNs [11],[12]. Both trials showed a high level of protective efficacy against symptomatic malaria (70% in Burkina Faso and 82% in Mali), together with reductions in moderately severe anaemia (56% and 48%, respectively) and severe malaria (69% and 87%, respectively). All children were given an ITN and were randomised to receive three doses, at monthly intervals, of SP plus AQ, or placebo, during the transmission season. Previous IPTc trials, in areas with seasonal peaks of transmission and low ITN use (<25%), have achieved similar efficacies of 69% with two doses of SP 8 weeks apart in Mali [9], 86% with three monthly doses of artesunate (AS) plus AQ in Senegal [8], and 69% with six monthly doses of AS plus AQ in Ghana [10]. Taken together, these findings suggest that IPTc would provide a valuable contribution in reducing malaria, by itself or integrated with other intervention strategies, in areas with highly seasonal malaria.

## New Evidence on the Delivery of IPT in Children

Few studies have examined the most efficacious and cost-effective means of delivering IPTc. Unlike IPTi and IPTp, which can be deployed as low-cost add-ons to existing frameworks, namely EPI (Expanded Program on Immunization) and antenatal clinics, IPTc does not have a similar existing mechanism. In this issue of *PLoS Medicine*, Bojang et al. now report important results from a randomised trial of two different modes of delivery [13]. Their findings suggest greater effectiveness in preventing symptomatic malaria when delivered by village health workers (VHWs) compared to reproductive and child health (RCH) trekking clinics. Delivery by VHWs achieved superior coverage, which was better sustained over the study period, and appeared to be more cost effective. The VHW arm had a tendency towards increased ITN usage and greater vaccination coverage. This further supports the important role of VHWs in malaria control in addition to delivering other health services to their communities, and broadly argues in favour of strong primary health care services in communities. While this model is potentially transferrable to other populations, each country considering IPTc implementation will face its own logistic considerations, in identifying, training, resourcing, and rewarding providers of IPTc.

Successful implementation of IPTc in different populations will need tailored drug selection that considers safety, tolerability, drug half-life, and cost, and specific regional circumstances, such as local patterns of drug resistance. Currently, SP/AQ appear to be favoured due to tolerability, efficacy, and affordability, but resistance to these drugs has reached alarming levels in many regions. Indeed, there was some evidence that markers of drug resistance increased in recent trials [11],[12], which probably reflects selection of pre-existing drug resistance; this may compromise the long term efficacy of these drugs. Presently, alternatives to SP and AQ are very limited. Because of the concerns over promoting drug resistance, it is preferable to use drugs in IPTc that are not used as first line for the treatment of malaria cases. The use of SP for treatment of malaria is being phased out in many countries, which may reduce the pressure for drug resistance.

The clinical benefit of IPTc in these recent studies indicates that implementation over a longer period of childhood would be appropriate. Administering IPTc for several years may have a substantial impact on acquired immunity such that a rebound in malaria may be a problem after IPTc ceases. At present, IPTc has been trialled as a short-term intervention (given during one season), and rebound was not observed [8]. The lifetime benefit of IPTc is likely to outweigh problems with rebound, and this potential problem should not be a barrier to implementing IPTc. Nevertheless, it will be important to anticipate and monitor these effects and consider how they might be mitigated.

## In What Settings Can IPTc Be Used?

With strong evidence that IPTc is effective in regions experiencing highly seasonal malaria, it remains to be determined at what level of malaria transmission and seasonality IPTc can be justified. It is also unclear how these factors should influence the age at which IPTc should be ceased. Determining the transmission level at which IPTc should be implemented, or continued in settings where malaria is declining, is an important priority. Similarly, the benefit of IPTc and how frequently it should be given in regions with extended malaria seasons or year-round transmission, or in populations outside Africa, is presently not clear. Studies to date have targeted *P. falciparum*, the major cause of malaria in Africa. However, in many regions *Plasmodium vivax* is an important cause of malaria, and further studies may be warranted to investigate whether IPTc could be used for this. The added complexity is that *P. vivax* has a dormant liver stage; clearing blood-stage parasites will not prevent further malaria, even in the absence of re-exposure.

## Conclusions

Based on the present evidence, we support the call for the implementation of IPTc as a population-level intervention in specific settings to reduce the burden of malaria in children. However, there is still much we need to know about when, how, and under what circumstances to implement IPTc and how to mitigate the potential impact of increasing drug resistance and impairing immune acquisition. These questions need to be a high priority for ongoing research, not only for IPTc, but alongside other interventions in a multi-faceted approach to malaria control.

## Abbreviations

AQ: amodiaquine
AS: artesunate
IPT: intermittent preventive treatment
IPTc: intermittent preventive treatment during childhood
IPTi: intermittent preventive treatment in infants
IPTp: intermittent preventive treatment in pregnancy
ITN: insecticide-treated bed net
RCH: reproductive and child health
SP: sulfadoxine-pyrimethamine
VHW: village health worker
WHO: World Health Organization
